# Supplementary material for: Are carbon emissions trading and green financial instruments synergistic? -Comprehensive quantitative research based on content analysis
Source: PLoS One. 2024 Mar 7;19(3):e0298601. doi: 10.1371/journal.pone.0298601 (PMC10919601; doi:10.1371/journal.pone.0298601)
Supplement: S1 Data — (ZIP) [file pone.0298601.s001.zip › Data/地级市Z_F工作报告.zip!/╔╜╢1⁄2/┴─│╟╩╨/└┤╫╘╣1⁄2╓┌║┼í╛┬φ┐╦╩2╛▌═°í┐_2019.docx]

各位代表：

现在，我代表市人民政府向大会报告工作，请予审议，并请市政协各位委员提出意见。

一、2018年工作回顾

过去的一年，是任务艰巨、压力倍增、爬坡过坎的一年，是励精图治、攻坚克难、努力拼搏的一年。在省委省政府和市委的坚强领导下，我们深入贯彻习近平新时代中国特色社会主义思想和党的十九大精神，坚定不移加快新旧动能转换，坚定不移推动高质量发展，坚定不移保障改善民生，始终保持政治定力、战略定力、发展定力，不图虚名，不骛虚声，着力化解长期积累的历史问题，破解制约发展的瓶颈问题，全年经济社会发展呈现稳中有进、进中提质的良好态势。

一年来，我们致力动能转换，发展质量持续提升。坚持把新旧动能转换作为经济工作的总牵引，全力推动质量变革、效率变革、动力变革。稳的基础更牢固。全年生产总值完成3152.2亿元，比上年增长5.4%；地方一般公共预算收入194.3亿元，增长4.2%，剔除不可比因素实际增长12.8%；规模以上项目投资增长22.8%；社会消费品零售额增长7.7%；进出口总额增长5.7%；居民人均可支配收入增长9.2%；居民消费价格上涨2.4%。进的势头更明显。突出表现在“五个快于”：三产增速快于生产总值3.1个百分点；高新技术产业产值增速快于规模以上工业产值5.8个百分点；税收收入增速快于一般公共预算收入3.9个百分点；居民人均可支配收入增速快于生产总值3.8个百分点；农村居民人均可支配收入增速快于城镇0.6个百分点。转的支撑更有力。在全省率先制定新旧动能转换实施规划和实施意见，设立了新旧动能转换基金，46个项目纳入省新旧动能转换项目库。建立市级“四新四化”项目库，储备项目202个，总投资3077亿元,开工159个。102个实施类市级大项目全部开工建设，完成投资345.2亿元。莘南高速建成通车，青兰高速工程建设任务过半，高东高速、市城区大外环征迁工作顺利完成，雄商高铁、郑济高铁、聊城机场等一批重大交通基础设施项目前期工作取得突破性进展。

一年来，我们致力创新驱动，产业结构持续优化。全市三次产业结构比例调整为9.9∶49.1∶41,三产占比首次突破40%,占比提高幅度居全省第2位。农业提质增效。以乡村振兴战略为统领，编制完成总体规划和五个单项规划，7个乡镇入选齐鲁样板示范乡镇,茌平耿店村“棚二代”创业实践得到习近平总书记高度肯定。持续实施产业化、科技化、组织化、品牌化“四化带动”，现代农业实现量质双升。粮食实现“十六连丰”，瓜菜菌总产达到1728万吨。建设“新六产”示范项目30个，创建现代农业产业园17处，全面建成11处高标准示范园区、10万个高效集约温室大棚。县级农业投入品集中配送中心全部建成使用，省级以上农产品质量安全县实现全覆盖，提前完成省定农药减量任务。东阿、茌平、高唐成功创建全国主要农作物全程机械化示范县。新增省级以上农业龙头企业14家，新增农民合作社865家、家庭农场275家。新认证“三品一标”112个、基地面积25万亩。“聊·胜一筹！”品牌进一步打响，每天直供京沪可追溯蔬菜达到200万斤，荣获2018中国区域农业品牌影响力第五名。工业转型升级。立足迈向产业中高端，加快推进产业集群化、产品高新化、模式新型化、品牌高端化，产业发展竞争力全面提升。全市100个工业重点项目完成投资187亿元，其中“延链、建链、强链”项目90个，完成投资172亿元。6家企业列为国家、省级“两化融合”管理体系贯标试点，新增上云企业763家。全年新增高新技术企业61家，创历史最好水平。新增省级以上创新平台18家、创新孵化企业22家。有效发明专利拥有量达1705件，增长12.5%。电气机械、医药制造、仪器仪表等先进制造业分别增长9.2%、12.2%和10.8%，全市高新技术产业产值占比提高1.7个百分点。服务业扩量提档。坚持把服务业作为结构调整的突破口，加快实施文化旅游业壮大、电子商务示范等六大工程，服务业增加值增长8.5%。京杭运河（聊城段）样板项目主体河道工程建设完成，东昌湖景区改造提升工程基本完工，中华水上古城旅游人数成倍增长，全市旅游消费总额增长15.2%。文化产业市场主体增长279%，增幅居全省第6位。引进光大银行、长江证券等金融机构7家，新三板企业总数达到33家。“无车承运人”等物流新模式蓬勃发展。质量强市深入实施。坚定不移推动质量强市战略，全国质量强市示范城市创建通过山东省预验收。主导或参与制定省级以上标准14项，新增地理标志商标5个、马德里注册商标7个、山东名牌27个。国家铜铝冶炼及加工产品质检中心通过验收，东阿阿胶、金号织业分别入选2018年最具价值中国品牌100强和500强。

一年来，我们致力攻坚突破，发展基础持续夯实。突出抓重点、补短板、强弱项，全力以赴打赢三大攻坚战。重大风险有效化解。全面开展金融风险排查，完善“一企一策、一企一预案、一企一专班”的工作机制，累计协调信贷资金近40亿元，处置不良贷款110亿元，有效化解部分重点企业风险，坚决守住了不发生区域性系统性金融风险的底线。加强政府债务管理，坚决遏制隐性债务增量，积极稳妥化解存量债务，彻底规范政府举债融资行为。精准扶贫成效显著。坚持党建筑基、精准施策、政策集成、责任包干，坚决打好脱贫攻坚战。整合财政涉农资金7.8亿元，累计发放扶贫贷款44亿元。“四大片区”共建成项目441个，完成投资近30亿元。实施产业扶贫项目132个,建成集中供养机构16处。全省乡村振兴暨脱贫攻坚西片区现场会在我市召开。0.93万建档立卡贫困人口全部脱贫，14.73万已脱贫人口保持稳定脱贫不返贫，全市脱贫任务基本完成。生态环境明显改善。始终把生态环保作为发展的“命门”，以“刮骨疗伤、壮士断腕”的决心推进生态治理，狠抓中央和省环保督察反馈问题整改，淘汰拆除落后燃煤机组21.2万千瓦。国家节能减排财政政策综合示范城市顺利通过验收。扎实实施“路长制”，扬尘治理整体向好。PM2.5、PM10、SO2、NO2均值浓度分别改善15.5%、11.1%、22.2%、2.5%，PM2.5均值浓度降至60微克/立方米，提前两年实现三年行动计划改善目标。优良天数176天，同比增加14天。获得省级生态补偿资金3812万元，居全省第3位。全面落实“河长制”“湖长制”。4个省控以上地表水考核断面均消除劣V类水体，8处集中饮用水水源地全部达到或优于Ⅲ类水质要求。大力实施森林进城围城等六大工程，成功创建为国家森林城市，成为全国平原城市“创森”典型。

一年来，我们致力统筹协调，城乡面貌持续改善。突出城乡一体、建管并重，加快建设中国北方高品质城市。完成住房城乡建设投资377亿元，11.5万农业转移人口和9.5万城中村、城边村居民实现市民化。规划体系更加健全。制定市中心城区近期建设规划、各县（市）总体规划和101个乡镇建设规划，启动全域水城总体规划编制，城乡规划不断优化。基础设施更加完善。棚户区改造开工4.9万套、基本建成2万套，改造老旧小区107个。新改建鲁化路、后菜市街等道路64条，新开工综合管廊10.5公里，新增海绵城市在建面积11.7平方公里。市城区新增供热面积170万平方米，110个小区实现直供到户。新建和提升各类公园147处，凤凰苑、二干渠花堤、周公河湿地等一批城市公园对外开放。望岳湖水库通过蓄水验收，我市8座南水北调水库已全部具备承接长江水能力。城市管理更加精细。大城管格局初步形成，城市管理效能明显提升。拆除违法建筑208万平方米，开展占道经营、流动摊点、露天烧烤专项整治，生活环境显著改善。实施中心城区交通优化提升工程，市区智能交通管控系统基本实现全覆盖，主干道通行率提高17.5%,百货大楼路口入选全国城市道路管理精选案例。村镇建设更加有力。第三批小城镇提升工程验收完工，创建省级美丽乡村示范村30个。启动农村人居环境整治三年行动，农村改厕任务基本完成。新建徒骇河、马颊河沿线污水处理设施25处，新增日处理能力1.8万吨。所有乡镇（街道）实现天然气管道全覆盖，完成农村清洁取暖13.6万户。新改建农村公路1361公里、危桥88座，农村公路建设三年攻坚行动提前完成。377个村完成“户户通”硬化路建设，建设里程1210公里。

一年来，我们致力改革开放，动力活力持续释放。着眼于增强发展内生动力，加速推进一批基础性、关键性举措落地落实。“放管服”改革全面深化。整合行政审批服务职能，组建市县行政审批服务局。推动政务系统信息互联和数据共享，实现电子政务市县乡全覆盖。推出“一次办好”市级事项1337项、县级平均1083项，“3545”专项改革目标基本实现。推出28项“菜单式”服务，实现了关联事项“一链办理”。“多证合一”扩大到“45证合一”，全市新登记各类市场主体增长13.1％。农村改革稳妥推进。农村集体产权制度改革全面铺开，5685个村基本完成改革任务。完善农村土地“三权分置”制度，农地抵押贷款金额达到4.6亿元。农村土地流转面积289万亩，占家庭承包总面积的39.7%。集体收入3万元以上的村增加714个，同比提高24%；空壳村减少759个，同比下降65%。双招双引成果丰硕。在京津冀、珠三角、长三角等重点区域举办了系列招商推介活动，全市招商引资到位资金347.1亿元，阿里云创新中心、智创未来轴承科技园、希杰万吨核苷酸等一批大项目、好项目成功落地。全市完成进出口总额489.6亿元，“一带一路”市场进出口额增长33.8%，连续6年入围全国外贸百强市。临清、莘县、阳谷获批国家级外贸转型基地。聊城海关正式获批挂牌。全市新增工程技术研究中心、科技孵化器、院士工作站等省级以上引资引智平台36个，“千人计划”“万人计划”专家、泰山学者等高层次人才51人。

一年来，我们致力共享发展，人民生活持续向好。始终践行以人民为中心的发展理念，扎实办好各项民生实事，民生财政支出324.2亿元，占一般公共财政预算支出的79.2%。教育事业加快发展。着力发展普惠性学前教育，新建、改扩建幼儿园100所。加快推进优质教育资源均衡化，新建、改扩建学校42所，全市中小学新招收班级均实现班额标准化。积极推动名校办分校，带动学校360所，惠及学生26万人。卫生改革深入推进。公立医院薪酬制度改革、按病种收费改革扎实开展。建立医疗联合体35个，覆盖医疗机构200余个。市人民医院、市第二人民医院、市中医医院顺利通过三甲复审。推进家庭医生签约服务，签约65岁以上老年人60万人。再次通过国家卫生城市复审，连续七届荣膺全国无偿献血先进市。社会保障不断增强。城镇新增就业7.2万人，技能培训2.9万人，城镇登记失业率控制在3.05%。在全省率先创建为国家社会保险标准化建设先行城市。全面提高了退休人员养老金、居民基础养老金、居民医保政府补助、城乡低保和失业救济金标准。文体事业更加繁荣。举办公益文艺演出4000余场，放映公益电影7.4万场，水城明珠保利剧场演出73场。市广电总台创作的电影《初心照黄河》获得公映许可。新建全民健身工程400处，实现乡镇(街道)、社区全覆盖。成功举办中国运河名城(聊城)自行车公开赛、“跑游山东”聊城半程马拉松等重要体育赛事。社会保持和谐稳定。“扫黑除恶”斗争取得阶段性胜利，打掉黑恶势力犯罪集团21个、恶势力团伙22个。建成市县乡村四级网格化综治中心，成功列入全国“雪亮工程”重点支持城市。生产安全事故起数、死亡人数分别下降16.9%和21.5%，是全省4个未发生较大以上事故的城市之一。食品安全抽检合格率97%以上，规模以上企业食品安全追溯体系覆盖率达到76%。全国文明城市创建取得积极成果，在全省新进提名城市测评中获得第1名。新闻出版、社会科学、民族宗教、港澳台、外事、侨务、地震气象、妇女儿童、青少年、老龄、对口支援等工作，都取得新的成绩。

同时，我们坚决贯彻全面从严治党的部署要求，严格落实中央八项规定精神，持之以恒纠正“四风”，切实推动政府系统作风转变，为全市经济社会发展营造良好的政务环境。忠实履行宪法和法律赋予的职责，坚持依法行政，自觉接受人大法律监督、工作监督和政协民主监督、社会舆论监督，共办理人大代表建议209件、政协委员提案325件，满意率和基本满意率达到99%以上。

各位代表，过去的一年，在新旧动能转换结构性问题凸显、长期积累的矛盾集中显现、经济运行困难增多的形势下，我们经受了考验，顶住了压力，成绩的取得来之不易。这是习近平新时代中国特色社会主义思想科学指引、掌舵领航的结果，是省委省政府坚强领导、亲切关怀的结果，是市委统筹全局、科学决策的结果，是人大、政协全力支持、有效监督的结果，也是全市上下攻坚克难、团结拼搏的结果。在此，我代表市人民政府，向全市人民，向人大代表、政协委员，向各民主党派、工商联、无党派人士和人民团体，向驻聊部队官兵和中央、省驻聊各单位，向所有关心支持聊城发展的海内外朋友，致以崇高的敬意和衷心的感谢!

各位代表，在看到成绩的同时，我们也清醒地认识到，我市在发展中还存在不少困难和问题，主要表现在：产业结构、能源结构不尽合理，发展的质量和效益还不高；新的经济增长点不够多，新旧动能转换步伐还不快；科技创新能力薄弱，高端人才相对匮乏；生态环境保护任务依然艰巨，个别领域金融风险压力较大；基本公共服务有待提升，上学、看病、出行等方面与群众需求还有不小差距；营商环境不够优化，少数干部不想为、不敢为、不会为、慢作为现象还不同程度存在。对此，我们一定高度重视，采取切实有效措施，认真加以解决。

二、2019年目标任务

2019年是新中国成立70周年，是全面建成小康社会的关键之年，也是我市加速新旧动能转换、推动高质量发展的攻坚之年。今年政府工作的总体要求是：以习近平新时代中国特色社会主义思想为指导，全面贯彻党的十九大和十九届二中、三中全会精神，深入落实习近平总书记视察山东重要讲话、重要指示批示精神，坚持稳中求进工作总基调，坚持新发展理念，坚持推动高质量发展，坚持以供给侧结构性改革为主线，坚持深化市场化改革、扩大高水平开放，以新旧动能转换重大工程为引领，加快实施创新发展战略，聚焦聚力基础设施建设、城市品质提升、乡村振兴、双招双引等重点工作，继续打好三大攻坚战，统筹做好稳增长、促改革、调结构、惠民生、防风险工作，着力构建现代化经济体系，为全面建成小康社会打下决定性基础，以优异成绩迎接中华人民共和国成立70周年。

综合考虑多方面因素，建议全市经济社会发展主要目标为：生产总值增长6.5%左右，地方一般公共预算收入增长5%左右，固定资产投资增长8%以上，规模以上工业增加值增长6.5%左右，社会消费品零售总额增长8.5%左右，进出口总额保持稳定增长，城乡居民人均可支配收入分别增长7.5%和8%左右，居民消费价格涨幅控制在3%左右；全面完成省下达的各类约束性指标。工作中，集中力量抓好以下九个方面：

（一）强力推进产业升级，培育高质量发展新动能。牢牢把握产业发展趋势和演进规律，实施产业集群建设工程，着力打造“5+4”产业集群，以更大力度推动存量变革、增量崛起。

全面提升五大优势产业集群。坚持把优势产业作为压舱石、稳定器，应用新技术、开发新产品、发展新模式，着力提升产业智慧化、智能化水平，确保主营业务收入、利税增长10%以上，增加值占生产总值比重提高0.5个百分点。集聚发展有色金属深加工产业，重点研发交通铝材、全铝汽车、精密铜箔等高端产品，加快推进信力源超薄电子铜箔、友升高端铝合金深加工等项目，延伸拉长产业链条，打造世界级高端铜铝加工基地。集聚发展绿色化工产业，重点建设鲁西聚碳酸酯、尼龙系列、华泰橡胶助剂等项目，加快莘县等化工园区提质升级，推动化工产业绿色高端转型。集聚发展纺织服装产业，突出智能化、绿色化、个性化方向，重点建设临清三和工业园、金号家纺科技产业园等园区，着力提升纺织服装产业产品档次和综合效益。集聚发展文化旅游产业，积极培育文化创意、动漫游戏、绿色印刷等文化融合新业态，加快京杭运河（聊城段）样板工程等项目建设，提升景阳冈、中洲古城等重点景区，推动东昌湖景区、东阿阿胶世界创建5A级景区，全力打造特色鲜明、充满魅力的旅游目的地城市。集聚发展现代高效农业，重点培植精品粮油、绿色蔬菜、生态林果、健康畜牧、特色水产五大特色产业，建设全国重要的绿色优质农产品供应基地。

培育壮大四大新兴产业集群。坚持把新兴产业作为新引擎、加速器，强化要素供给，突破核心技术，确保主营业务收入、利税增长15%以上，增加值占生产总值比重提高1个百分点。加快发展新材料产业，重点发展稀土永磁、碳化硅等产品，全力推进上达稀土、齐鲁生物基材料、波米科技等重点项目，尽快形成规模效应。加快发展先进装备制造产业，重点推进金帝精密机械、诺伯特工业机器人、鑫泰机床机器人等项目，推动东昌府保持器、临清轴承等重点园区转型升级，增强产业创新能力和市场竞争力。加快发展新能源汽车产业，重点推进通洋氢燃料电池、中通智能网联客车、时风电动商用物流车等项目，狠抓核心关键零部件的研发配套，打造全国重要的新能源汽车生产基地。加快发展医养健康产业，依托聊城医疗资源和阿胶产业优势，重点建设市医养中心、伏城医养健康产业园、东阿阿胶国家中医药健康旅游示范基地等项目，支持社会力量兴办医养结合机构，实现医疗、医药、养老、养生等多业态融合发展。

健全强有力的保障推进机制。实行重大项目市级统筹，统一规划用地、统一明确布局，实现资源高度集中、要素优化配置。建立重大项目集体决策机制，对重点产业龙头项目和投资50亿元以上的大项目，按一事一议的方式确定扶持政策。实行“五个一”工作机制，每个产业集群配套一个发展规划、一名帮包领导、一个工作专班、一个科研机构、一笔专项资金，全力推进产业集群建设。

（二）强力推进创新驱动，增强高质量发展新动力。在爬坡过坎、滚石上山的重要关口，我们必须增强发展定力，顶住转型阵痛，以更大决心、更大力度推进“脱胎换骨、破茧成蝶”。一是深入推动投入扩增。坚定不移地实施大项目带动战略，依靠项目做优产业、做强经济。加快实施100个新旧动能转换项目，确保完成年度投资350亿以上。着力推进100个先进制造业项目，全面提高工业发展竞争力。持续实施服务业“双50”工程，抓好50个重点项目，培育50户龙头企业。切实优化投资结构，确保高新技术产业投资增幅高于固定资产投资。二是深入推动技术引领。瞄准高端化，利用三年时间，对规模以上企业实施新一轮技术改造，完善政产学研金服用合作机制，统筹设立九大产业集群研究院，新增省级以上创新平台16家、高新技术企业20家，研发投入占生产总值比重达到2.2%左右。瞄准智能化，发展智能工厂、网络协同制造、大规模个性化定制等新业态新模式，加快智汇谷·阿里云创新中心、瑞柏生物、蓝天七色智能制造等重点项目建设。瞄准绿色化，加快培育绿色示范园区、绿色工厂、绿色设计产品，推动电解铝、化肥、氯碱等高耗能行业绿色转型，确保通过国家循环经济示范城市验收。三是深入推动质量提升。实施“标准化+”行动，主导或参与修订各级标准10项以上。实施“万千百十”品牌培育计划，新注册商标5000件以上，加快建设阳谷特种光电线缆、临清特种专用轴承全国知名品牌示范区。实施质量提升行动，优化鲁西质检中心检验检测认证服务体系，建成国家轴承产品质检中心，支持东阿阿胶争创中国质量奖、太平洋光纤光缆等争创省长质量奖，巩固提升全国质量强市示范城市创建成果。四是深入推动观念变革。大力开展“转型再造·升级蜕变”行动，倡树新时代企业家精神，支持企业主动参与新旧动能转换重大工程。注重转型发展，坚决摒弃产品层次低、质量效益差、污染排放重的粗放发展模式，选准方向和路径，研发新产品、拓展新市场。注重机制创新，引导企业家大胆推进管理变革，打破家族式管理模式，引进战略投资者，建立现代企业制度，敢于在管理体制、股权改造、资本运作、人才激励等方面实施突破。注重社会责任，增强大局意识，坚守环保、安全、能耗、用地等底线红线，确保既能做大、更能做优，争做受社会尊敬、让员工自豪的百年企业。

（三）强力推进基础设施建设，筑牢高质量发展新支撑。抢抓国家和省发展战略机遇，全力推进基础设施建设。一是集中攻坚交通建设。充分发挥冀鲁豫三省交界区位优势，全力打造区域性综合立体交通枢纽城市。开工建设雄商高铁、郑济高铁，积极推动聊邯长（青兰）高铁前期工作，加快形成纵贯南北、横连东西的“十”字型高铁大通道。争取聊城机场预可研报告获得国家批复，力争2020年开工建设。建成青兰高速聊城段，加快高东高速建设进度，推动德州至高唐高速、聊城至鄄城高速、德上高速临清连接线等项目前期工作，加快实现高速公路成环成网、互联互通。力争聊泰铁路黄河大桥、东阿至东平黄河大桥全面开工，加快推进G105东阿黄河大桥前期工作，打通跨越黄河的快速通道。抓好G309冠县段等干线公路建设，营造“畅、安、洁、绿、美”的公路通行环境。二是持续推进水利建设。全面落实水安全保障总体规划，集中力量形成大建设、大发展的强劲态势。加快彭楼灌区改扩建工程建设，实施位山、彭楼、陶城铺灌区续建配套节水改造工程，推动大型灌区引黄渠首闸改建前期工作。实现望岳湖水库引蓄长江水，加快南水北调配套水厂和供水管网工程建设。着力抓好徒骇河、马颊河防洪治理，开工建设王堤口、刘桥、王铺等中型水闸除险加固工程，彻底消除两岸群众水患之忧。三是全面优化能源建设。加快城乡电能改造升级，新建改造变电站49座、线路1157公里、变电容量368万千伏安。实施非化石能源倍增行动计划，重点推动莘县生物质发电、冠县和高唐垃圾发电等项目，支持清洁低碳能源优先上网。加快武城—阳谷、道口铺—冠县、新奥等天然气管道建设，新增天然气管道182公里，新增输气能力10亿立方米，进一步提高供应质效。四是统筹抓好信息建设。建设市大数据共享管理平台，加快构建“数字政府”。打造“互联网+民生服务”平台，实现一个APP慧享水城。加快城市骨干网络扩容升级，新建基站1600个，新铺设高速光纤光缆3000公里。建设市云计算中心（二期）项目，规划鲁西大数据生态产业园，抓好互联网、大数据与传统产业深度融合。

（四）强力推进城市建设，构建高质量发展新格局。加快建设高品质现代化区域中心城市，力争用五年时间，市中心城区人口突破200万人，建成区面积突破200平方公里。一是实施空间优化工程。采取“中疏、东进、北拓、南展、西优”的空间发展策略，提升中心城市功能。实施聊茌东都市区空间发展战略规划，实现茌平撤县设区，适时启动东阿撤县设区，建设市域副中心，拓展城市发展空间。规划建设古漯河城市绿心，加快构建聊茌东快速绿色交通廊道。谋划启动高铁新城建设，突出科技创新、金融服务、高铁物流等功能，打造产业新高地、生态智慧城。加快推进开发区、高新区、度假区产城融合，实现城市组团式发展。以东昌湖、望岳湖联结廊带为轴线，打造代表水城形象的集中展示区，建设高品质城市会客厅。二是实施功能提升工程。立足全域水城建设，实施运河东昌湖周公河水系连通、徒骇河南北延伸、班滑河水系连通等一批重点项目，勾画出河湖相济、湖网相连、人水相依的美丽画卷。完善城市道路体系，全市新改建城镇道路103条、167公里。加快实施122公里的市城区大外环工程，建设中华路、黑龙江路跨河大桥，推进湖南路西延、西关街西延、二干路北延等重点项目，着力构建城市发展交通新格局。提升城市绿化水平，按照“300米见绿、500米见园”要求，建设玉皇皋公园、卫育紫荆公园、东昌工业遗产公园、东昌文化广场等一批城市公园、绿地广场，确保九州洼月季公园“五一”建成开园，更好地满足市民休闲游赏的需求。三是实施城市更新工程。分类分区制定城市更新策略，逐步降低主城区居住用地比例，增加公共绿地和开敞空间。全面优化城市设计，精心打造万达欢乐小镇、国际金融中心等街区和路段，建设精致城市。严格保护中华水上古城历史格局和风貌肌理，复兴和延续文化传承功能，妥善保护米市街、大小礼拜寺街等历史文化街区，推进优秀历史建筑的活化利用。加快城市修补、生态修复，改造提升青年渠、小湄河、四新河等河流景观，实施站前南街、昌润路、陈口路等雨污分流改造工程。建设山东省装配式建筑示范城市。四是实施治理提质工程。开展全国文明城市创建活动，全面提升城市文明程度。深入推进乱搭乱建、乱停乱放、占道经营、农贸市场环境等专项治理，还城市以整洁、给市民以方便。深入研究改善城区交通的系统方案，适时启动地上地下立体交通项目，开展文明畅通提升行动，下大气力解决重点区域、重点路段交通拥堵问题。着力完善住房供给和保障体系，规范稳定房地产市场。深化城市管理体制机制改革，逐步推进清扫保洁、市政维护、园林绿化等运营市场化，提升城市管理精细化水平。

（五）强力推进乡村振兴，夯实高质量发展新基础。聚焦聚力产业、人才、文化、生态、组织“五大振兴”，高质量推进农业农村现代化，努力走出一条具有聊城特色的乡村振兴之路。

一是立足提质增效，发展现代农业。强化产业支撑。着力稳定粮食、蔬菜产量，确保粮食产能保持在100亿斤以上、蔬菜总产值突破200亿元。稳定生猪等畜禽生产，做好非洲猪瘟等疫病防控。实施30个农业新旧动能转换重点项目，深度开发食用菌、大豆、肉鸡、黑毛驴等重点产品，加速延伸绿色食品产业链条。积极推进三产融合发展，重点打造“新六产”示范项目30个，创建美丽生态牧场50处、部级水产健康养殖示范场10处，建设一批有特色、有效益的田园综合体。强化科技引领。加强与中国农科院、中国农大等高校院所的产学研合作，完成重大科技成果转化10项。高标准建设莘县国家农业科技园区和10处市级以上现代农业产业园，推动莘县、东阿争创国家级现代农业产业园，打造现代农业发展样板。积极推进“两全两高”农机化建设，农作物综合机械化率达到89%以上。强化组织带动。大力培育新型农业组织，新增规模以上农业龙头企业30家、农民合作社500家、家庭农场100家，新建提升为农服务中心20家。鼓励新型经营主体与农户建立契约型、股权型利益联结机制，推动农户与现代农业有机衔接。发挥聊城职业技术学院等院校的专业优势，培训新型农民2000人。强化品牌提升。推动农业投入品集中配送体系向乡镇（街道）、村（居）延伸。加快构建管理有制度、操作有标准、生产有记录、质量可追溯的农业标准化体系，“三品一标”认证面积占比达到55%以上。持续推动“净菜进京入沪”工程，组建“聊·胜一筹！”品牌运营公司，引进京东、阿里、北菜、上农等电商平台和重点企业建立直采直供基地，在京津沪济建设一批品牌旗舰店、专营店，大力拓展大中城市高端市场。

二是立足生态宜居，建设美丽乡村。强化美丽乡村建设顶层设计，实行一次规划、分步实施，推进生活、生产、生态“三生共融”。按照农村新型社区、农业产业园区、乡村旅游景区“三区联建”的思路，沿河、沿湖、沿路和重点企业周边实施乡村振兴示范项目20个以上，建设一批产业特色村、传统文化村、红色记忆村和乡村旅游村。实施农村人居环境整治行动，建立改厕管护长效机制，94%的村庄实现生活垃圾无害化处理，农作物秸秆综合利用率达到91.5%，持续巩固提升城乡环卫一体化成果。推进“四好农村路”建设，新改建农村公路620公里，改造危桥44座。建设省级美丽村居示范点6处。

三是立足强基固本，深化农村改革。推进农村集体产权制度改革，确保90%以上的涉农村（居）完成改革任务。完善农村土地所有权、承包权、经营权“三权分置”制度，扎实开展农地抵押贷款试点，稳慎推进宅基地所有权、资格权、使用权分置改革试点。推动农业保险提标扩面，开展温室大棚和小麦全成本保险试点。扶持壮大村级集体经济，基本消除空壳村。实施“归雁”工程，鼓励各类人才返乡创新创业。探索实行绩效管理机制，激发村（居）干部群众干事创业的热情。

四是立足精准扶贫，巩固脱贫攻坚成效。加快发展特色扶贫产业，加强养驴扶贫、光伏扶贫的运营管理，建好县级扶贫项目库，实现所有权、经营权、收益权、监督权分置管理。积极探索政府支持和商业保险相结合的扶贫模式，撬动更多社会资金参与扶贫攻坚。聚焦特困群体，新建、改扩建一批集中供养中心，推广邻里互助模式，推进教育、医疗、住房等保障性扶贫政策与社会保障制度相衔接。强化动态管理，对因病因灾出现的临时性、突发性、支出性贫困人口，即时发现、即时帮扶。开展遍访贫困对象行动，确保帮扶措施精准落地。在全面建成小康社会的伟大征程中，我们将与全市人民守望相助、砥砺同行，决不让一个贫困群众掉队！

（六）强力推进各领域改革，激发高质量发展新活力。以深化政府机构改革为契机，全力推进重点领域改革攻坚。一是深化“一次办好”改革。规范行政审批服务局运行，实行“一枚印章管审批”。优化审批流程，推进“一窗受理”“一链办理”，实现市县两级100个高频事项“一次办好”。同步建设实体大厅和网上大厅，市县两级政务服务事项网上可办率达到70%以上。加快实现部门政务信息横向互联、市县乡服务联审平台纵向贯通。二是激发国资国企活力。积极引进高质量战略投资者，鼓励社会优质资本参与国企改革，稳妥发展混合所有制经济。健全务实管用的国有企业激励考核机制，确保国有资产保值增值。加快财金公司、财信公司、土储集团、旅发集团等国有公司市场化改革，选准主业，做大做强。落实国有资产集中统一监管，将县属经营性国有资产全部纳入监管体系。三是防范化解金融风险。持续做好政府债务管控，严格规范政府举债行为，做到态度坚定、处置有序、整体可控。全面落实重点企业金融风险化解方案，稳妥有序推进地方金融机构风险化解和转型发展，严厉打击恶意逃废债务行为和各类非法金融活动，处置不良贷款60亿元，确保风险不外溢扩散。大力推进企业公司制改制，确保50%的规模以上企业完成改制任务。鼓励企业对接多层次资本市场，培育上市后备企业10家，重点推进凤祥集团、嘉华股份、哈鲁轴承、乖宝宠物食品等企业上市进程，实现直接融资100亿元以上。培育壮大地方金融机构，着力打造普惠性金融体系。四是全力支持民营经济发展。落实好惠企政策，全面贯彻中央和省支持民营经济高质量发展的各项部署，不折不扣落实减税降费政策，开展营商环境突出问题专项整治，切实降低企业制度性交易成本。服务好民企需求，制定重点行业、重点企业精准帮扶措施，着力解决项目用地、融资需求、技术攻关、手续办理等实际困难。推动好民企转型，实施“专精特新、育苗扶壮”工程，新增省级“一企一技术”企业6家以上，培育更多行业“隐形冠军”和“瞪羚企业”。构建好政商关系，研究制定政商交往正面清单和负面清单，坦荡真诚与企业家交往，始终把企业家作为社会的宝贵财富，始终与企业家同心同向、风雨同舟，始终坚定支持企业家一心一意干事业、心无旁骛谋发展！

（七）强力推进双招双引，打造高质量发展新高地。树立大开放观，深度融入京津冀协同发展区等重大战略，以更广阔的视野、更包容的姿态、更坚定的步伐，扩大对外合作，加快借势发展。一是引进新项目。聚焦高端终端产品，重点引进产业链条延伸型项目。聚焦技术创新前沿，重点引进关键设备、核心零部件等支撑型项目。聚焦传统产业转型升级，重点引进新技术、新模式、新业态等融合型项目。深入研究世界500强、中国500强、民营500强投资方向和区域布局，量身定制合作项目，确保新增九大产业集群过亿元开工项目100个以上，全市招商引资到位资金300亿元以上。二是汇聚新智慧。以顶尖人才、高端人才、紧缺人才和成长性人才为重点，依托聊城大学等地方高校，力争引进高层次专家人才350名。实行党委政府联系服务专家制度，在家属安置、子女教育、住房保障、医疗保健等方面不断改进服务措施。启动新旧动能转换服务中心和市级人才公寓建设。持续办好“院士专家聊城行”“海外高层次人才交流会”等活动，擦亮引智引才品牌。建设更多孵化器、创客空间，支持大学生、海归人员等年轻人创新创业。三是打造新载体。制定出台建设高铁沿线产业隆起带的指导意见，沿郑济高铁谋划布局茌平新材料、东阿阿胶养生、高铁新城创新中心、莘县高效农业等四大园区，沿雄商高铁谋划布局临清高端装备制造、世界水城、阳谷光电等三大园区。突出开发区差异化、特色化发展，重点引进投资过10亿元的产业引领项目，打造一批集聚度高、竞争力强的产业集群，大幅提高新上项目亩均投资强度和亩均效益。做好聊城开发区、东阿开发区全省体制机制创新试点工作，力争聊城高新区创建为国家级高新区。四是拓展新市场。紧紧抓住“一带一路”重大机遇，重点推动有色金属、纺织、化工、钢铁板材、机械制造等优势产业“走出去”，支持优质企业开展海外并购，参与国际合作。整合优化物流产业资源，加快建设聊城内陆港、保税物流中心、铁临物流园、百佳美国农产品运营仓储中心等项目。五是完善新机制。实行重点区域招商常态化机制，定期开展综合性招商推介活动。推动政府引导与市场化招商相结合，拓展委托招商、代理招商等招商渠道。加强乡缘招商引才，引导拥有较强实力、掌握先进技术、业界影响力大的聊城籍人才回聊投资发展。深入开展“二次招商”，支持内外资企业增资扩股、新上项目。建立更加有力的常态化督导机制，提高项目开工率和资金到位率。

（八）强力推进生态保护，厚植高质量发展新优势。坚决扛起生态文明建设的政治责任，狠抓中央、省环保督察反馈意见和“回头看”移交问题整改，深入推进“四减四增”专项行动，加快建设天蓝、地绿、水清的美丽聊城。一是铁腕“减煤”。开展燃煤锅炉和工业炉窑综合整治，基本淘汰35蒸吨／小时以下燃煤锅炉，其余锅炉全部完成节能和超低排放改造。积极推进冬季清洁采暖，坚持以气定改、以电定改，新增清洁能源采暖10万户。积极推广电能替代燃煤，力争电量替代10亿千瓦时。二是精准“治污”。加大工业大气污染综合整治，对重点行业开展挥发性有机物深度治理。着力控制柴油货车污染，实施国六排放标准。推进鲁西化工、信发、冠洲、大唐临清等重点企业，新建或改建铁路专用线，减少公路运输量。深入实施“路长制”，严格落实“五个三”扬尘治理措施。PM2.5平均浓度控制在60微克／立方米，空气质量优良率达到53.3%。三是系统“治水”。扎实推进“河长制”“湖长制”，全面加强河湖管理保护工作。完成徒骇河水质提升工程，确保水质全面达标，省控以上河流断面达标率达到75%。实现所有建制镇污水处理设施全覆盖，确保污水处理率达到60%以上。四是综合“净土”。加快聊城市固体废物综合利用处置项目建设。推进有机肥替代化肥，确保到2020年单位耕地面积化肥使用量比2015年下降6%。强化病虫害绿色防控，全市农药折百使用量控制在1200吨以下。推动畜禽集中养殖区建设，畜禽废弃物利用率达到80%以上。五是全域“绿化”。巩固提升国家森林城市创建成果，完善城乡绿色生态系统，确保新增造林面积7万亩，林木绿化率提高0.5个百分点。

（九）强力推进民生改善，共享高质量发展新成果。坚持以人民为中心的发展思想，尽力而为、量力而行，着力补齐民生短板，不断提高城乡群众的获得感、幸福感、安全感。

推动社会事业全面进步。坚持教育优先发展。落实第三期学前教育行动计划，着力解决入园难、入园贵的问题。持续加大义务教育投入，资源更多向农村中小学倾斜。调整市城区义务教育阶段公办、民办学校结构，增加公办学校在校生比例。深入推进县管校聘、校长职级制和绩效管理等改革，精准施策提高义务教育质量。加快建设现代职业教育体系，推进办学体制、人才培养模式等改革，深化产教融合、校企合作。健全社会保障体系。提高城乡低保标准、优抚对象补助标准、失业救济金标准，落实经济困难老年人补贴、残疾人补贴扩面提标制度。加大临时救助和医疗救助力度，将符合条件的困难群众全部纳入社会救助保障范围。提高孤儿和困境儿童基本生活费补助标准，切实维护妇女儿童权益。大力整顿工程建设市场秩序，从源头上整治拖欠农民工工资问题。深化健康聊城建设。启动市级健康促进示范县（区）建设，支持阳谷、东阿创建省级健康促进示范县。充分发挥三级医院的引领作用，积极组建城市医疗集团。加快县级医院能力建设，推动县域医共体全覆盖。全面提升基层卫生院医疗水平，力争基层医疗机构标准化建设全部达标。提升双拥共建水平。做好退役军人移交安置和服务管理工作，严格落实优抚政策，维护军人军属合法权益，激励他们在新时代建功立业。积极争取中央财政对革命老区的专项补助，推动刘邓大军强渡黄河战役纪念馆列入国家红色旅游经典景区。维护社会安定和谐。加强风险分级管控和隐患排查治理体系建设，强力推动安全标准化达标升级，坚决遏制较大以上安全事故。抓好建国70周年安保维稳工作，打好公安基层基础攻坚战，深入开展“扫黑除恶”专项斗争，加快“智慧公安”和“雪亮工程”建设，做好普法教育、信访稳定等工作，确保社会大局和谐稳定。认真做好新闻出版、广播电视、社会科学、气象地震、民族宗教、体育、档案、外事、侨务、港澳台、对口支援等工作。

全力办好十件民生实事。一是新增城镇就业4.5万人以上，其中失业再就业1万人以上、困难群体就业2500人以上。二是新建、改扩建普惠性幼儿园50所，新建中小学11所、增设班级160个，新增学位7600个。三是棚户区改造开工8332套、基本建成6029套，改造老旧小区40个。四是加快发展居家养老、居家护理，新建社区老年人日间照料中心17处。五是做实家庭医生签约服务，确保65岁以上老年人签约率稳定在70%以上，做到签约一人、履约一人。六是优化市城区公交线路，投放共享型公共自行车1万辆、增设站点500个。七是丰富群众文化生活，推进县乡村三级新时代文明实践中心全覆盖，在水城明珠保利剧场举办高水平文艺演出50场以上。八是开展物业服务提升行动，推进老旧小区物业管理全覆盖。九是全市新增集中供热面积300万平方米，市城区直供到户率达到70%以上。十是坚决打赢农村饮水安全攻坚战，今明两年解决3112个村庄、239万人的饮水安全问题，实现城乡“同网、同源、同质”和全部达标。民之所望，政之所向。只要我们认真倾听群众呼声、用心回应群众关切，一件接着一件办,一事接着一事干,就一定能让人民群众的生活更加幸福美满！

三、加强政府自身建设

习近平总书记指出：伟大梦想不是等得来、喊得来的，而是拼出来、干出来的。我们将深入学习贯彻习近平新时代中国特色社会主义思想，保持斗争精神、增强斗争本领，以“工作落实年”为总抓手，把各项工作向深处做、向细处做、向实处做，努力开创政府工作新局面。

（一）突出忠诚履职，增强凝聚力。始终忠诚于党，扎实开展“不忘初心、牢记使命”主题教育，树牢“四个意识”，坚定“四个自信”，做到“两个维护”，确保政令畅通、令行禁止。始终忠诚于人民，坚持问政于民、问计于民、问需于民，聚焦经济社会发展的痛点、堵点，围绕群众关切的热点、难点，谋对策、办实事，做到“有求必应、无事不扰”。始终忠诚于职责，强化“以能力比高低、以实绩论英雄”的鲜明导向，及时准确地将市委部署转化为举措，咬定目标、攻坚克难，真正在善为上有办法，在敢为上勇担当。

（二）突出解放思想，增强创新力。境界决定标准，标准决定质量。推动新一轮思想大解放，着力解决思维固化、标准不高、效率低下等问题，以思想破冰引领行动突围。全面开展寻标对标活动，各项工作都要在全省乃至全国寻找坐标和定位，找出差距、列出清单，自我加压、争创一流。实施干部队伍专业素质提升工程，重点加强产业、金融、环保、安全等方面专业培训，提升谋划工作、推动发展的能力。构建务实有效的制度体系，强化正向激励，大胆容错纠错，让勇于担当者有底气，让实干有为者有奔头。

（三）突出作风转变，增强执行力。推动落实是政府一切工作的生命线。这能力那能力，不落实就等于没能力。突出领导干部带头，当好“施工队长”，大力践行“一线工作法”，做到点对点掌握情况，面对面解决问题。着力提高政府效能，坚持立说立行、速决速行、雷厉风行，做到“案无积卷、事不隔夜”。建立闭环督办机制，推行“责任定位法”，对重大部署、重要工作定目标、定标准、定时限，做到“事事有着落、件件有回音”。认真落实中央、省关于减轻基层负担的部署要求，研究出台具体实施办法，大幅减少会议、材料、督导、考核，决不能让高高在上的官僚主义助长“重痕不重绩”的形式主义。合理界定“属地管理”适用范围，完善主体责任和配合责任清单，出台关心关爱基层的配套制度，加大力度继续提高基层干部待遇，切实解决值班、就餐、下村等实际保障问题，充分调动基层干事创业的主动性和创造性。

（四）突出依法施政，增强公信力。法定职责必须为，法无授权不可为。大力弘扬法治精神，把依宪执政、依法行政贯穿于政府工作全过程，严格依照法定权限和程序履行职责。深化综合行政执法改革，加快推进职能、权限、程序、责任法定化。自觉接受人大的法律监督、工作监督和政协的民主监督，认真听取各民主党派、工商联、无党派人士和人民团体意见。全面推进政务公开，加强政务诚信建设，畅通社会监督和媒体监督渠道，让权力在阳光下运行。

（五）突出廉洁从政，增强约束力。党员干部应当知足知不足、有为有不为。坚决扛牢全面从严治党主体责任，认真履行“一岗双责”，全面落实意识形态责任制。深入贯彻中央八项规定精神，持之以恒纠“四风”、正作风。深化审计管理体制改革，强化脱贫攻坚、公共资金、国有资产、公共资源交易等领域的监管，注重审计和巡视巡察结果运用。坚持过“紧日子”，严控 “三公”经费和一般性支出。严肃查处侵害群众利益的突出问题，深化和巩固反腐败斗争压倒性态势。持续开展廉政教育，使遵规守纪成为根植内心的素养、无需提醒的自觉。

各位代表！实干托起梦想，奋斗铸就辉煌。让我们更加紧密地团结在以习近平同志为核心的党中央周围，在省委省政府和市委的坚强领导下，着力凝聚加快发展的聊城共识，汇聚团结奋斗的聊城力量，彰显攻坚克难的聊城担当，锐意进取、狠抓落实，为新时代开启聊城发展新征程、谱写聊城发展新篇章而不懈奋斗！
